# Foreword - Kidney disease in disadvantaged populations: an unconquered challenge 

**DOI:** 10.5414/CNP83S001

**Published:** 2015-02-23

**Authors:** Kowdle S. Prabhakar, Keith C. Norris, Lawrence Agodoa, Guillermo García-García

**Affiliations:** 1 Singapore; 2 Los Angeles, CA,; 3 Bethesda, MD, USA, and; 4 Guadalajara, Mexico

**Keywords:** kidney disease, disadvantaged populations

## Abstract

No abstract available.

This online supplement summarizes the presentations that were made at the 2013 World Congress of Nephrology Satellite Symposium “Kidney Disease: A Global Challenge of Epidemic Proportions,” the 9^th^ meeting organized by the Committee for Kidney Health in Disadvantaged Populations, an advisory committee of the International Society of Nephrology (ISN CKHDP). The primary mission of the Committee is to explore ways to reduce the burden of kidney disease in disadvantaged communities in both developing and developed countries, including racial and ethnic minority groups that suffer disproportionately from kidney disease and its complications. 

This symposium was held from June 4 to June 5, 2013 at the Hong Kong Academy of Medicine in Aberdeen, Hong Kong, immediately following the biennial World Congress of Nephrology (from May 31 to June 3, 2013). The presentations covered a diverse range of nephrology topics with emphasis to disadvantaged populations. Most of the topics presented in the sessions are further elaborated in the manuscripts published in this supplement of Clinical Nephrology. 

In summary, there were stimulating presentations about novel early detection and prevention programs from several countries, susceptibility factors contributing to progression of CKD, genetic and environmental factors accounting for unique regional nephropathies, availability and affordability of renal replacement therapy for the unfortunate patients who reach end-stage renal disease (ESRD), and issues with organ allocation for the lucky few who have access to transplantation. 

This being the 9^th^ installment of the satellite meetings, ISN CKHDP has come a long way in highlighting the unique problems faced at least in some of the developing countries as well as disadvantaged communities within the developed countries. This satellite has seen growing interest among the physicians, researchers, and policy experts as evidenced by the increasing number of delegate attendance and abstract submission. 

Has there been an improvement in the care of “disadvantaged populations”, what has been done and where are we today? Unfortunately, the early detection programs in most of these economically challenged countries are either not available or very limited and fragmented, not adequately validated and with the data analyzed/derived, there seems to be no meaningful implementation process. In many countries, the burgeoning incidence and prevalence of CKD has overwhelmed the support for proper follow-up and management to retard its progression. Support for ESRD care is mostly economically driven, truncated also by limited trained or qualified personnel. 

Patients from the resource-limited countries continue to be disadvantaged with very limited access to general healthcare, restricted access to recommended prevention and early intervention at various stages of CKD, and limited ESRD support. Further, many patients continue to be exposed to toxins and pollutants, which account for clustered cases of regional nephropathies. In addition, limited access to potable water and proper sewerage contributes to much of the infection-related renal diseases. 

Where do we go from here and what can be done to improve the situation? Primary impetus would be to educate the public at large and create the awareness regarding hypertension, diabetes mellitus, and CKD. ISN and IFKF joining hands to spearhead awareness programs across the globe especially on “World Kidney Day” is one such effort to create awareness. Governments and policy makers on health issues and other stake holders should focus on providing safe drinking water, good sanitation, and creating awareness on hygiene at large and improve early detection and prevention programs in particular. Unless there is unrestricted free access to healthcare for the impoverished, no healthcare program focused on alleviating the suffering of people will be successful. In tandem, every effort should be made to train doctors, nurses, and other healthcare providers with basic knowledge and infrastructure to implement these programs successfully. While financing these will be a difficult task in the emerging countries, every developed nation should join hands to help; probably by forming a “global health fund” and use the funds rightly to reach the beneficiaries in greatest need, who are the patients. In addition to having its great outreach programs, ISN should train more doctors and such training programs should be tailor-made to suit the basic needs of impoverished countries. Most importantly, doctors and the healthcare personnel having equipped with proper training, should be committed to serve their respective parts of the world, instead of migrating to developed countries seeking greener pastures. 

It is indeed a herculean task to conquer this unmet challenge of caring for the disadvantaged populations around the world, but joint efforts should definitely lead us in the right direction. The ISN CKHDP has been playing its role by linking together people from the developed and the developing world who strive to care for the disadvantaged population, at the same time facilitating the exchange of ideas for providing renal care in resource-limited geographical areas. Growing interest in this arena with every installment of the satellite is a big step forward. 

